# CIS (change impact score) – a novel outcome measurement tool to quantify the relevance of medical education interventions on professional performance

**DOI:** 10.1080/21614083.2017.1375377

**Published:** 2017-09-18

**Authors:** L. Taatz, V. Wenzel, G. J. Peiseler

**Affiliations:** ^a^ Medical Department, OmniaMed Deutschland GmbH, Munich, Germany

**Keywords:** CME (continuing medical education), CIS (change impact score), outcome assessment, education quality, physician performance

## Abstract

Outcome measurements play a key role in professional CME (Continuing Medical Education). While assessment of delegate satisfaction and knowledge transfer is a common standard, it appears desirable to address higher levels of evidence. However, measurement of competence and performance is considered complex, difficult and expensive. The CIS (Change Impact Score) is a novel instrument to predict the relevance of the educational intervention to the professional performance of the physician, based on a standardised on-site self-assessment.

## Introduction

All physicians practising in Germany are legally obliged to participate in CME (Continuing Medical Education). Every five years a total number of 250 CME credit points must be collected and submitted to the Association of Statutory Health Insurance [1]. Each credit equals 45 minutes of professional medical education. The content needs to be certified by the relevant medical association (chamber of physicians) of the federal state.

On account of these legal requirements and in general over the last decade, CME has become more and more important and beyond that highly sophisticated [2,3].

The evolution of medical education has not only been influenced by academic standards but is in addition increasingly professionalised [4–7].

OmniaMed is an independent provider of CME for health professionals offering a variety of live education formats and digital modules for general practitioners, various medical specialist groups and pharmacists. Each year, approximately 50,000 CME credit points are distributed via OmniaMed. As one of the leading CME providers in Germany, OmniaMed feels obliged to drive education performance, using a complex quality management system. National and international exchange, e.g. within the European CME Forum and the Good CME Practice Group, is considered to provide best practice insights and trigger further development of CME tools such as outcome measurements.

Current evaluation methods for CME events usually consist of questionnaires which inquire the quality of the speaker, the quality of the content and the overall satisfaction with the CME event. However, those evaluations are limited since a speaker might be highly qualified but the content of the talk may not be translated into every-day practice. At the same time, there is no direct feedback to the speakers concerning the relevance of the talk to daily practice. Therefore, a method is required to evaluate the relevance of the talk which enables the CME organiser directly to compare the impact of different topics on everyday practice.

## Method

The current medical content quality management includes a mandatory medical review of every single presentation in order to ensure the compliance with the medical standards of the OmniaMed Institute [8].

On site evaluation of the programme is also mandatory for all OmniaMed formats. So far, this evaluation is based on paper, using a standardised evaluation form (see Appendix 1). This form asks for the delegate satisfaction with scientific validity, content neutrality, speaker know-how and presentation skills, as well as organisational and administrative aspects.

Another mandatory tool is a knowledge transfer assessment after each live or digital education module. This is also paper-based and complies with the requirements of the relevant chamber of physicians.

With reference to the 7 Levels of CME Outcome Measurements, established by Donald E. Moore [9,10], the OmniaMed outcome assessment so far covers the first three levels: participation, satisfaction and learning. In order to aim at level 4 (competence) and level 5 (performance), although potentially biased because future procedures can only be predicted by a delegate’s self-assessment, we developed a standardised evaluation of the expected impact of the individual learning module on the delegate’s professional performance: CIS (Change Impact Score).

The CIS is a standardised on-site measurement tool analysing the relevance for the daily practice of the physicians participating in the live event.

**Figure 1.**
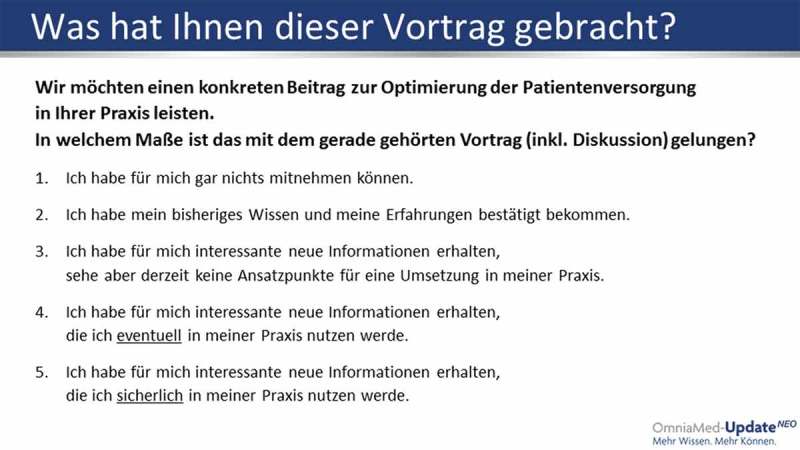
CIS Question. Original CIS Evaluation Chart

During the live conference or other possible formats (e.g. workshops, round tables, etc.), the delegates are asked to answer a standardised question after each presentation/discussion via an audience response system. The question asks for the expected relevance of the medical content to their professional performance with five options to answer (Figure 1– CIS Question):

- no relevance to knowledge or performance
- confirmation of current knowledge and performance
- new and relevant information but no expected impact on professional performance
- new and relevant information with potential impact on professional performance
- new and relevant information with definite impact on professional performance

**Figure 2.**
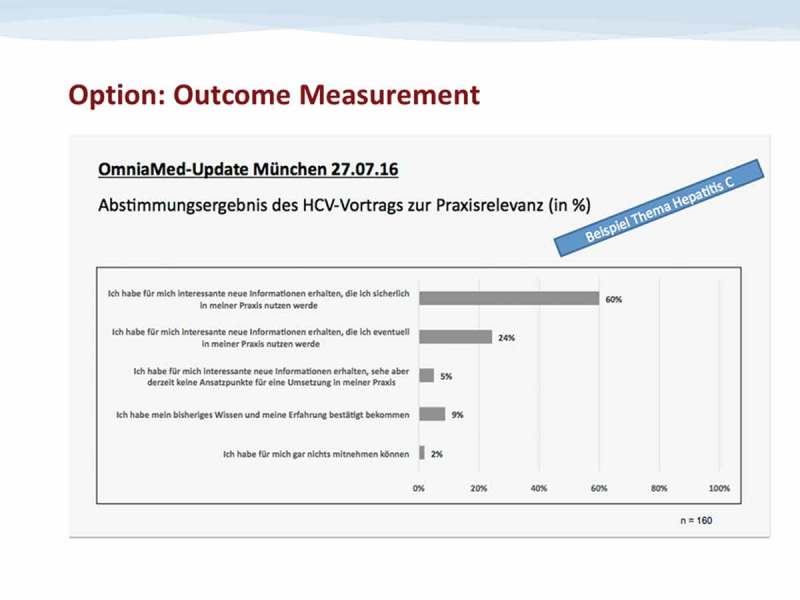
Voting results. Exemplary Voting Result (referring to a lecture on Hepatitis C during a GP education meeting in Munich on 27.07.16, when CIS as a novel outcome measurement tool was tested for the first time)

The results are displayed immediately to the audience and to the speaker, using a 5 bar matrix indicating the voting result (percent of votes for the 5 options) (Figure 2– Voting results).

To further evaluate the survey, the answers are correlated with a score from 0 (= no relevance to knowledge or performance) to 4 (= new and relevant information with definite impact on professional performance).

**Figure 3.**
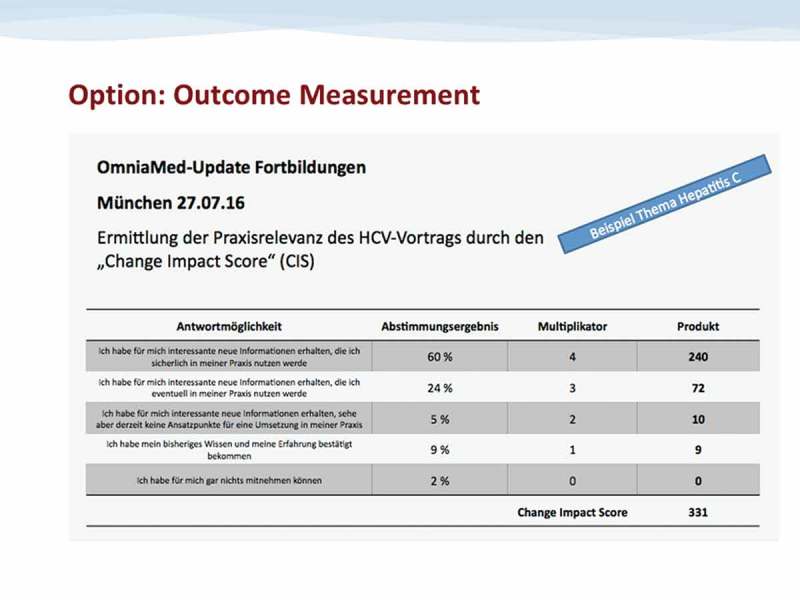
Derivation of final CIS. Calculation of the CIS Value (shown for the exemplary lecture on Hepatitis C in Munich on 27.07.16)

The percentage of the voting results is multiplied with the assigned factor (0–4) (Figure 3– Derivation of final CIS). The resulting 5 figures (one being 0 by definition) are summed up providing the CIS which is defined as a unit-free measure:

$$\begin{array}{l} CIS=\left(\frac{Nvote_{0}}{Ntotal}*100\right)*score_{0}+\left(\frac{Nvote_{1}}{Ntotal}*100\right)*score_{1}\\ +\left(\frac{Nvote_{2}}{Ntotal}*100\right)*score_{2}+\left(\frac{Nvote_{3}}{Ntotal}*100\right)*score_{3}\\ +\left(\frac{Nvote_{4}}{Ntotal}*100\right)*score_{4} \end{array}$$

CIS = Change Impact Score

N_total_ = Total number of participants

N_vote(x)_ = Number of participants who voted per answer x

Score_x_ = Score (0–4) that is assigned to the answer x

The potential maximum CIS is a score of 400 which reflects the case that a hundred percent of the participants voted “new and relevant information with definite impact on professional performance.” The potential minimum CIS is a score of 0 which reflects that a hundred percent of the participants voted “no relevance to knowledge or performance.”

**Figure 4.**
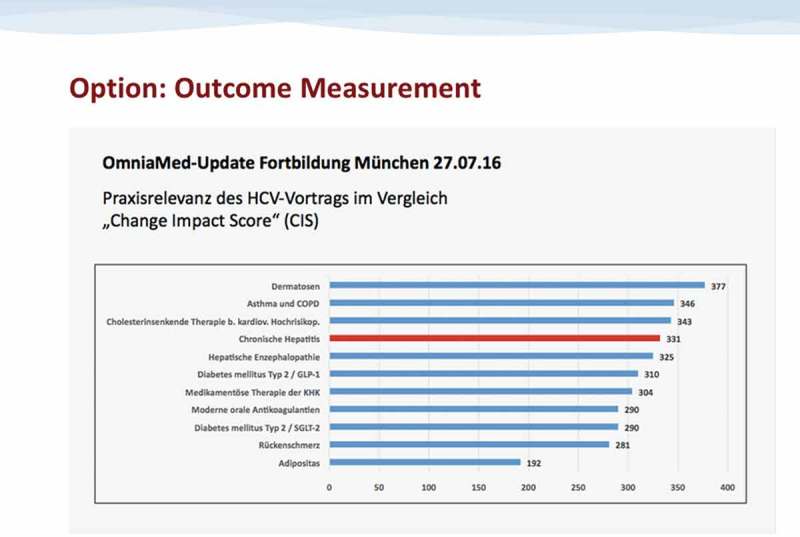
Exemplary CIS results. CIS Values Obtained For the Lectures During the Test Education Meeting in Munich on 27.07.16 (exemplary lecture on Hepatitis C marked in red)

First tests performed during live education meetings in Q3/Q4 2016 showed a fairly consistent range of CIS measurements between 280 and 350, indicating sufficient reliability of the method (Figure 4– Example CIS results).

The CIS will be implemented during a series of live medical education events (OmniaMed Update^NEO^ events) throughout Germany with an expected total of approximately 4,000 physicians in 2017 (1 year observation period)

## Expected results

The CIS concentrates the expected potential of an educational intervention (e.g., presentation) to influence professional performance into a simple three-digit figure. This figure can be used as follows:

- Quality Attribute: Obviously, presentations with high CIS are more impactful to the audience. Speakers will aim at achieving a high CIS score, especially when it is displayed to the public on site.
- Quality Management Tool: By comparing the CIS amongst different presentations during a live meeting, those with lower CIS can be identified as requiring further work-up on either content or didactics or both.
- Quality Monitoring: By adding up all CIS figures of an individual meeting, it will be possible to compare the overall change impact of individual educational events, and the individual range of minimum/maximum CIS per meeting may be another quality indicator.
- Speaker Monitoring: By comparing the CIS of a presentation given by an individual speaker several times, it will be possible to see whether he/she is gaining or losing his/her ability to motivate the audience for professional development
- Speaker Training: By comparing the CIS of different presentations given by different speakers on the identical subject, it will be possible to identify the speaker with the most impactful presentation, thus providing a basis for sharing best practice.

Another expected result is a systematic feed-back on the mandatory medical review process: In advance of the events all presentations are reviewed by the medical team of OmniaMed in a standard procedure. In this process OmniaMed uses specific guidelines [8] in order to guarantee a high content quality. Besides a check for scientific good practice, the medical review also contains an examination of grammar, didactic structure, transparency, neutrality and an estimation regarding the relevance for the daily practice of physicians. With the CIS measurement, it will be possible to give a validated feedback to the speakers in order to improve the relevance of their future lectures.

In summary, the CIS can be used to monitor the quality of medical content, as well as the development of the speakers over time, and it is expected to give feedback on the internal medical review process.

Due to the easy application of the CIS – as an add-on to questionnaires aiming at quality of scientific content, rhetoric performance, etc. – it can be used for every kind of CME-live-event evaluation such as conventions, workshops, seminars, focus groups or to evaluate the impact of digital CME-measures such as webinars, eCME modules or mobile applications. However, the limitations of the CIS are that it is on-site and based on self-assessment of the physician. Both facts tend to rather overestimate the effect on future professional performance. This will have to be taken into account when the results of the 2017 series are analysed.

## Conclusion

The CIS may represent a useful performance indicator for high quality in CME. It provides a measurable value of the impact of the educational programme on daily practice with various implications on quality management and speaker development. Beyond that it offers the opportunity to improve the medical review process. Based on these characteristics, the CIS may contribute to increasing the overall quality of CME.

The results of CIS measurement during the 2017 OmniaMed Update^NEO^ series will be analysed and we will share our experience with the CME community in a follow-up report in early 2018.
